# Use of kanamycin for selection of *Eucalyptus saligna* genetically transformed plants

**DOI:** 10.1186/1753-6561-5-S7-P147

**Published:** 2011-09-13

**Authors:** Yohana Oliveira, Lais Adamuchio, Juliana Degenhardt-Goldbach, Isabel Gerhardt, João Bespalhok, Roberson Dibax, Marguerite Quoirin

**Affiliations:** 1 UFPR , Brazil; 2 EMBRAPA-Florestas, Brazil

## Background

Several factors may affect the genetic transformation efficiency of woody species. One factor is the use of an efficient selective agent that inhibits the development of non-transformed cells and just allows the development of transformed tissues. The most used selection agent is the neomycin phosphotransferase II (*NPTII*) gene, which confers resistance to aminoglycoside antibiotics kanamycin, neomycin and G-418 [1].

The selective agent concentration in culture medium may have influence on shoot regeneration and high concentrations may promote adverse effects on organogenic potential [2]. The kanamycin effects in *Eucalyptus* are variable and depend on the species and genotypes [3]. The purpose of this study was to evaluate the effect of kanamycin concentration on transformation efficiency for *Eucalyptus saligna* cotyledons after co-culture with *Agrobacterium tumefaciens*.

## Methods

The bacterial strain was EHA 105, containing a binary vector carrying the *GUS* gene under control of CaMV35S promoter and *NPTII* gene under control of the same promoter. Cotyledons from twelve days old *E. saligna* plantlets were co-cultured for 30 min in the bacterial suspension (OD_600nm_ = 0.5) followed by a 5 day co-culture on MS culture medium containing 2.7 µM NAA + 4.4 µM BAP in the dark. The explants were then transferred on the same medium supplemented with (1) 12.5 mg L^-1^ kanamycin (Km) + 300 mg L^-1^ Augmentin (Aug); (2) 25 mg L^-1^Km+ 300 mg L^-1^ Aug and (3) 50 mg L^-1^Km+ 300 mg L^-1^ Aug. The explants were subcultured in the same culture medium every 15 days and, after 60 days, the percentage of oxidation, callus and shoot formation, and shoot number per explant were evaluated. DNA was extracted from fresh young leaves and processed according to the specific protocol [4]. The presence of *GUS* gene in the putative transformed plants was confirmed by PCR 10 months after inoculation.Gus expression was studied byhistochemical test for ß-glucuronidase, three and 120 days after inoculation.

## Results

With regard to the percentage of oxidation, percentage of callus formation and percentage of shoot formation, there was no significant difference among the three treatments. The percentages of explants regenerating shoots were 24, 15.9 and 14.6 respectively for the three Km concentrations, after 60 days of co-culture period. The lower concentration of kanamycin (12.5 mg L^-1^) showed best results for shoot regeneration (24%) and number of shoots per explant (3.9) and these results were statistically differents of those obtained with other treatments. Three gus positive events were regenerated from explants cultured on medium containing 12.5 mg L^-1^Kmand the transformation efficiency was 0.0075 %.

## Conclusion

The concentration of 12.5 mg L^-1^Kmallowed the shoot induction from genetic transformed tissues and was considered satisfactory for selection of transformed tissues. The information presented here may constitute the basis for optimization of the genetic transformation of other *E. saligna* genotypes.

## Acknoledgements

The authors thank CNPq (Conselho Nacional de Pesquisa, Brazil) and Fundação Araucaria (Sciences Secretary, Parana State, Brazil) for funding the research.
